# Teachers’ Duties

**Published:** 1885-04

**Authors:** 


### TEACHERS' DUTIES

Those teachers who have in charge children with very little opportunity of *home* training, may probably notice what is being done for such pupils in English schools, as shown in an article from the *Sanitary Engineer.* We suppose farmers' boys and girls to have the advantage of practical training at home from necessity: but that there is need of enlightenment even in the farm-houses is shown by the following remarks from one of our Western subscribers, an observant woman:

"I have of late been thinking considerably of what knowledge is most useful to women, and I have come to the opinion that most of our public institutions of learning do not even recognize the information that we need most, while some branches that are almost entirely unused in after years are taught at a great expense. A large proportion of our women raise children and have the care of households; to them the knowledge that will help them to do this well is of the first importance. Any mother would exchange her knowledge of reading and writing to save the life of her child, yet girls will learn everything else before the care of children; and consequently many children die from no other cause than their mothers' ignorance. It is supposed that girls will learn all that is necessary of this kind of information from their mothers, but most mothers are not competent teachers, having only learned of *their* mothers, and so on through generations. I'll mention a few mistaken ideas common to housekeepers here, and I see no hope of their ever being corrected: They say it is a sure sign their canned fruit is keeping well when a mold has formed over the top; that yeast is sour when the flour settles to the bottom and the water rises rises to the top; that very hot water will set dirt in clothes and they can never afterward be made clean—I mean if it be put on them when dry. One smart young girl was sure soda would cause gas bubbles in sweet milk, as in sour. An intelligent lady who has expended hundreds of dollars on her musical education, used on her table tomatoes that had fermented sufficiently to burst the jug in which she had sealed them. Ill-made clothing and badly cooked food are very common, while the waste of time and energy in poor methods of doing work cannot be estimated."

The young men and women who teach most of the country schools cannot be expected to remedy all this, since the best way to learn how to do things is to *do* them; but the practical applications of many scientific facts could be pointed out, and young minds trained to compare and reason, and so become able to apply principles for themselves.—*The Student,*

